# Family resilience mediates exposure to adverse childhood experiences on insufficient sleep among children: findings from a population-based study

**DOI:** 10.3389/fped.2024.1281599

**Published:** 2024-06-27

**Authors:** Philip Baiden, Christian E. Vazquez, Catherine A. LaBrenz, Fawn A. Brown

**Affiliations:** ^1^ School of Social Work, The University of Texas at Arlington, Arlington, TX, United States; ^2^ Department of Psychology, The University of Texas at Arlington, Arlington, TX, United States

**Keywords:** family resilience, family functioning, adverse childhood experiences, insufficient sleep, adolescents

## Abstract

**Background:**

Sleep plays a vital role in the well-being of children and adolescents. Researchers have identified adverse childhood experiences (ACEs) as an important factor associated with poor sleep among adolescents. The objective of this study was to examine the mediating role of family resilience on the association between ACEs and insufficient sleep among adolescents in the United States.

**Methods:**

Data for this study came from the 2018–2019 National Survey of Children's Health (*N *=* *28,097). The outcome variable in this study was insufficient sleep, and the main explanatory variable was exposure to ACEs. The mediating variable was family resilience. Data were analyzed using binary logistic regression.

**Results:**

Based on parent reports, one in five (22.4%) adolescents did not meet the recommended sleep hours on an average night. About half of the adolescents had no ACEs, 24.2% had one ACE, and 14.6% had three or more ACEs. Controlling for the effect of other factors and family resilience, the odds of having insufficient sleep were 1.63 times higher for children exposed to three or more ACEs (AOR* *=* *1.63, 95% CI* *=* *1.30–2.05). Family resilience partially mediates the association between exposure to ACEs and insufficient sleep. Each additional increase in family resilience decreased the odds of having insufficient sleep by a factor of 12% (AOR* *=* *0.88, 95% CI* *=* *0.86–0.91).

**Conclusions:**

Family resilience partially mediated exposure to ACEs on insufficient sleep. There are modifiable factors that may improve sleep outcomes among adolescents who have been exposed to adversity. Future research can help elucidate findings and establish the directionality of this association.

## 1 Introduction

Sleep plays a vital role in maintaining the health and well-being of children and adolescents (1). The United States (U.S.) National Sleep Foundation considers sufficient sleep as 9–11 h for children aged 6–13 years, and 8–10 h for adolescents aged 14–17 years (2). For the purposes of this study, adolescents are defined as children between the ages of 10 and 17 years. Various studies have found that most adolescents in the U.S. are not getting the recommended hours of sleep (1, 3). For instance, Kann et al. (3) examined data from the 2017 Youth Risk Behavior Survey (YRBS) and found that only one in four adolescents in the U.S. got the recommended eight or more hours of sleep on an average school night.

Insufficient sleep among adolescents has been found to be associated with daytime sleepiness, falling asleep during class, conduct problems at school, poor academic performance, and substance use (1, 4, 5). Evidence tying insufficient sleep to poor health outcomes such as obesity and mental health problems among adolescents is also growing (6). In contrast, sufficient sleep has been associated with positive mental health and school achievement during adolescence (1). Addressing insufficient sleep among adolescents is critical to preventing these adverse outcomes from continuing into adulthood. One potential factor that has received less attention in the sleep literature is family resilience as a mediator of exposure to adversity on sleep. For the purposes of this study, family resilience is defined as a family's ability to thrive and function within the context of adversities (7). Family resilience is grounded in both structural and developmental perspectives thereby allowing for a holistic approach to family functioning. A recent systematic review found that positive family relationship was associated with better sleep quality, whereas distressing family relationship and social isolation were associated with poor sleep quality among children and adolescents (8). However, the review by Gordon et al. (8) calls for additional studies to understand the link between family relationships and sleep quality among vulnerable populations.

Various studies have been conducted to understand factors associated with insufficient sleep among adolescents. One important factor that has been identified as associated with insufficient sleep among adolescents is a history of trauma and, in particular, adverse childhood experiences (ACEs) (8–11). ACEs are defined as stressful experiences (e.g., parental divorce, parental incarceration) that occur prior to age 18 that negatively impact health throughout the life course (10). Available research indicates that about one in three individuals in the U.S. has experienced at least one ACE (12). Recent research suggests that ACEs-related health outcomes may begin earlier than previously identified (13). Using data from 817 families from the Fragile Families and Child Wellbeing Study, Rojo-Wissar et al. (10) found that ACEs at age 9 were significantly associated with more social jetlag and higher odds of trouble falling asleep, after adjustment for symptoms of anxiety, depression, and demographic factors. Santos et al. (14) also examined data on adolescents aged 10–17 years from the 2016–2018 National Survey of Children's Health (NSCH) and found that adolescents with two or more ACEs were significantly less likely to meet the recommended screen time and more likely to have inadequate sleep.

Family resilience has been found to mediate the pathway between exposure to ACEs and school engagement (15) and mental health problems (16). However, less is known about how family resilience could mediate the association between exposure to ACEs and insufficient sleep among adolescents in the U.S. Thus, examining the mediating role of family resilience on the association between ACEs and insufficient sleep among adolescents is warranted. The current study focuses on adolescents between the ages of 10–17 years and uses a strengths-based perspective in the operationalization of family resilience given that family resilience literature suggests that using the family as a unit of resilience shows how the family adapts and changes to flourishing during stressful periods (17).

Research in the area of resilience provide ample evidence demonstrating that family resilience is associated with flourishing, even among adolescents exposed to ACEs (13, 18). For example, one study found that school engagement was higher among adolescents exposed to ACEs but had higher family resilience (15). Gómez (19) also found that families that regularly engage in mutual support and problem-solving may be better positioned to promote mental and physical health outcomes across generations. With respect to the association between resilience and sleep, Sivertsen et al. (20) examined data from 9,338 adolescents aged 16–19 year from Norway and found that compared to adolescents in the no delayed sleep phase (DSP) group, adolescents in the DSP group scored significantly lower on all subscales on the resilience measure, including personal structure, personal competence, family cohesion, social support, and social competence. Chang et al. (21) also examined data on 2,280 adolescents in northern Taiwan and found that family dysfunction was negatively and significantly associated with both resilience and sleep quality. In contrast, higher levels of resilience were significantly associated with better sleep quality. They also found that, controlling for other factors, resilience significantly mediated the effects of all family dysfunction trajectories on sleep quality. Taken together, the extant literature suggests that examining how family resilience can buffer the adverse effect of exposure to ACEs on insufficient sleep may help in the development of interventions for adolescents exposed to ACEs (8).

### 1.1 Adolescent and family vulnerability as covariates of interest in the current study

Considering the suggestion from the literature to understand sleep within the context of diverse types of family dynamics, there are several factors that reflect adolescent and family vulnerability that are included in the current study. These factors include: immigrant status, household poverty level, receipt of welfare assistance, child has special healthcare needs, functional difficulties, and body-mass-index (BMI). These factors have been associated with ACEs, sleep, or family resilience and can reflect increased vulnerability due to financial needs (e.g., exposure to poverty, receipt of public assistance) or potential for exposure to discrimination (e.g., immigrant status) (22). Immigrant status has been found to be associated with insufficient sleep, although this pattern varies by race/ethnicity (23). Additionally, one study found that children from immigrant families had lower odds of ACEs despite extreme poverty (24). Family resilience approaches have been proposed for immigrant families (25), and this provides the rationale for examining various pathways between the main variables of interest. Literature linking household poverty and sleep is well documented, with the majority of the literature indicating that poverty and receipt of welfare assistance are associated with insufficient sleep among adolescents (26). Similarly, children with special needs or disabilities tend to have worse outcomes related to sleep (27). Lastly, BMI has been associated with worse sleep and ACEs among adolescents (28). The literature linking all these factors provides the rationale for including a comprehensive set of covariates to understand the association between exposure to ACEs and insufficient sleep.

### 1.2 Current study

Although research has examined the association between ACEs and sleep among adolescents (11), few studies in the U.S. have examined the mediating effect of family residence on the association between ACEs and insufficient sleep among adolescents. Drawing on a large nationally representative sample of adolescents aged 10–17 years in the U.S., the objective of this study was to investigate (1) the association between ACEs and insufficient sleep, and (2) the mediating effect of family resilience on the association between ACEs and insufficient sleep. Based on the extant literature, we hypothesized that: (1) ACEs would be positively associated with insufficient sleep, (2) family resilience would be inversely associated with insufficient sleep, and (3) family resilience would mediate the association between ACEs and insufficient sleep.

## 2 Data and methods

### 2.1 Data source and participants

The data used for this study came from the 2018–2019 National Survey of Children's Health (NSCH) conducted by the U.S. Census Bureau on behalf of the U.S. Department of Health and Human Services, Health Resources and Services Administration, and Maternal and Child Health Bureau. Detailed information about the NSCH, including the objectives, methodology, and sampling procedure, is provided in its methodology report (29, 30) and other publications by the authors (31–33). In brief, the NSCH is a nationally representative survey designed to: (1) estimate national and state-level prevalence for a variety of child and family health measures; (2) generate information about children, families, schools, and neighborhoods to help guide policymakers, advocates, and researchers; and (3) provide baseline estimates for federal and state performance measures, Healthy People 2020 objectives, and state-level needs assessments (29).

The 2018–2019 NSCH covers topics such as demographics, health and functional status, health care access and utilization, early childhood (0–5 years) issues, issues specific to middle childhood and adolescence (6–17 years), family functioning, parental health status and family, and neighborhood and community characteristics. The 2018–2019 NSCH covered children ages 0–17 years who live in households nationally and in each state. For households with more than one child, one child per household is randomly selected to be the subject of the detailed age-specific topical questionnaire. There were 59,963 children and adolescents ages 0–17 years in the 2018–2019 NSCH. However, the analyses presented in this study focuses on adolescents aged 10–17 years old (*N* = 28,097). The respondents to the 2018–2019 NSCH are parents or guardians who best know the child's health. However, given that NSCH reflects the population of children and adolescents ages 0–17, not parents or families, we report the findings in terms of children/adolescents, even if the question refers to the parents or family (e.g., if the parent reports living in poverty, we report on the child living in poverty). The 2018–2019 NSCH data have been de-identified and are publicly available; hence, no institutional review board approval was required.

### 2.2 Variables

#### 2.2.1 Outcome variable

The outcome variable investigated in this study is insufficient sleep and was measured as a binary variable. Parents were asked, “DURING THE PAST WEEK, how many hours of sleep did this child get on an average weeknight?” The American Academy of Pediatrics endorsed a guideline developed by the American Academy of Sleep Medicine (34). The guideline recommends the following sleep hours: children aged 6–12 years should sleep 9–12 h a day, and children aged 13–18 years of age should sleep 8–10 h a day. For the purposes of this study, adolescents who met the recommended guideline were coded as 0, whereas adolescents who did not meet the recommended guideline were coded as 1.

#### 2.2.2 Explanatory variables

The main explanatory variable examined in this study was exposure to ACEs. Exposure to ACEs was measured based on primary caregiver reports. Primary caregivers were asked, has this child EVER experienced any of the following?: (1) hard to get by on your family's income, (2) parent or caregiver divorced or separated, (3) parent or caregiver died, (4) parent or caregiver served time in jail, (5) saw or heard parents or adults slap, hit, kick punch one another in the home, (6) was a victim of violence or witnessed violence in the neighborhood, (7) lived with anyone who was mentally ill, suicidal, or severely depressed, (8) lived with anyone who had a problem with alcohol or drugs, and (9) treated or judged unfairly due to race/ethnicity. Each ACE item was coded 1 if the child has ever experienced this form of adversity, and no = 0, if the child had not experienced this form of adversity. A count measure of ACEs was then created by summing each item to arrive at the total number of ACEs with scores ranging between 0 and 9, with higher scores indicating exposure to more ACEs. Due to the non-normal distribution of ACEs scores, three or more were combined into one category and treated as an ordinal variable in the analysis (0, 1, 2, and ≥3). This measure of exposure to ACEs has been used in previous studies (31, 35).

#### 2.2.3 Mediator

The mediator variable examined in this study was family resilience. Family resilience was measured using four items that asked parents, “When your family faces problems, how often are you likely to do each of the following”: “talk together about what to do”, “work together to solve our problems”, “know we have strengths to draw on”, and “stay hopeful even in difficult times”. Response to each item was measured on a Likert scale ranging from 1 “none of the time” to 4 “all of the time”. Scores on the family resilience measure range from 4 to 16, with higher scores indicating higher family resilience. The family resilience measure has been used in past research and has been found to be a valid measure of family resilience with strong internal consistency (15). In the present study, internal consistency (Chronbach's α) for the family resilience measure was *α* = 0.89.

#### 2.2.4 Covariates

Other covariates examined in this study included immigrant status, household poverty level, receipt of welfare assistance, child has special healthcare needs, functional difficulties, and BMI. Immigrant status was measured as a binary variable based on parents' response to the question, “Was this child born in the United States?” Children born in the U.S. were coded as 0, whereas children born outside the U.S. were coded as 1. Household poverty/income level was measured based on the Federal Poverty Level (FPL) and was coded into the following categories “0 = 0%–99% FPL”, “1 = 100%–199% FPL” “2 = 200%–399% FPL”, and “3 = 400% or above FPL”. The FPL is calculated as the ratio of total family income and the family poverty threshold and reported as a rounded percentage. The family poverty threshold is derived from the Census Bureau's poverty thresholds. Thresholds vary by family size and the number of related children under 18 years. Receipt of welfare assistance was a composite measure based on response to the following four survey items that ask about whether someone in the child's family received: (1) benefits from the Woman, Infants, and Children (WIC) Program, (2) cash assistance from government welfare program, (3) Food Stamps or SNAP benefits, or (4) free or reduced-cost breakfasts or lunches at school during the past 12 months. Children with special health care needs were identified using the children with special health care needs screener, which is a five-item parent-reported tool designed to reflect the federal Maternal and Child Health Bureau's consequences-based definition of children with special health care needs. Children with special health care needs were coded as 1, and children with no special health care needs were coded as 0. Functional difficulties were measured based on responses to a series of questions that asked: “Does this child have one or more functional difficulties from a list of 12 difficulties?” Following the recommendation of the NSCH (29), children who do not have any functional difficulties were coded 0; children with one functional difficulty were coded 1, and children with two or more functional difficulties were coded 2. BMI in adolescents is age and sex-specific. BMI for age and sex categories was categorized into “Normal = under 85th percentile”, “Overweight = 85th–94th percentile”, and “Obese = 95th percentile or greater”.

#### 2.2.5 Demographic variables

The following demographic variables were taken into account. Age was coded into 10–12 years, 13–15 years, and 16–17 years. Child sex was coded into “0 = Female” and “1 = Male”. Lastly, race/ethnicity was coded into “0 = non-Hispanic White”, “1 = non-Hispanic Black”, “2 = Hispanic”, and “3 = Other race/ethnicity”.

### 2.3 Data analyses

Descriptive, bivariate, and multivariate analytic techniques were employed in analyzing the data. The general distribution of all the variables included in the analysis was first conducted using percentages for categorical variables and mean and standard deviation for family resilience. This was followed by a bivariate association between insufficient sleep and the categorical variables using Pearson Chi-square test of association. The multivariate analysis involves the use of binary logistic regression to examine the association between exposure to ACEs and insufficient sleep while adjusting for family resilience and other covariates. Three logistic regression models were fitted. In Model 1, we regressed insufficient sleep on demographic and covariates. Model 2 consists of variables in Model 1 plus exposure to ACEs. Model 3, which is the fully adjusted model consists of variables in Model 2 plus family resilience. Adjusted odds ratios (AOR) are reported together with their 95% Confidence Intervals (C.I.). Variables were considered significant if the *p-*value was less than .05. Stata's “svyset” command was used to account for the weighting and complex survey design employed by the NSCH. All analyses were performed using Stata 17 M.P.

## 3 Results

### 3.1 Sample characteristics

Table 1 shows the general distribution of the variables examined in this study. Based on parent reports, we found that a little over one in five (22.4%) children did not meet the recommended sleep hours on an average night. Based on parent reports, about half of the children had no ACEs, about one in four (24.2%) had one ACE, 10.6% had two ACEs, and 14.6% had three or more ACEs. Family resilience was negatively skewed with an average resilience score of 13.5 (SD = 2.5). The sample was evenly distributed by sex (male = 50.6%). A little over half (51.3%) of the children were non-Hispanic White, with the remainder identifying as non-Hispanic Black (13.1%), Hispanic (25.5%), and Other (10.1%). A little over 15% of children received welfare assistance, and about one in four (24.6%) had some special health care needs. A little over two-thirds (68.9%) of the children had no functional difficulties, 18.8% had one functional difficulty, and 12.3% had two or more functional difficulties. About 70% of the children were classified as underweight/normal, 15.4% overweight, and 15.4% as having obesity.

**Table 1:** Descriptive statistics (*N* = 28,097).

Variables	Frequency (%)
Outcome variable	
Insufficient sleep	
No	21,807 (77.6)
Yes	6,290 (22.4)
Explanatory variable	
ACEs	
None	14,214 (50.6)
One ACE	6,794 (24.2)
Two ACEs	2,978 (10.6)
Three or more ACEs	4,111 (14.6)
Demographic and covariates	
Resilience, mean (SD, range)	13.5 (2.5, 4–16)
Age	
10–12 years	10,626 (37.8)
13–15 years	10,506 (37.4)
16–17 years	6,965 (24.8)
Sex	
Male	14,231 (50.6)
Female	13,866 (49.4)
Race/ethnicity	
Non-Hispanic White	14,419 (51.3)
Non-Hispanic Black	3,683 (13.1)
Hispanic	7,166 (25.5)
Other race/ethnicity	2,829 (10.1)
Immigrant status	
No	26,670 (94.5)
Yes	1,427 (5.1)
Household poverty level	
0–99% FPL	4,836 (17.2)
100–199% FPL	6,162 (22.0)
200–399% FPL	8,015 (28.5)
400% FPL or greater	9,084 (32.3)
Receipt of welfare assistance	
No	23,810 (84.7)
Yes	4,287 (15.3)
Child has special health care needs	
No	21,183 (75.4)
Yes	6,914 (24.6)
Functional difficulty	
None	19,350 (68.9)
One functional difficulty	5,286 (18.8)
Two or more functional difficulties	3,460 (12.3)
BMI	
Underweight/Normal	19,438 (69.2)
Overweight	4,339 (15.4)
Obese	4,320 (15.4)

ACEs, adverse childhood experiences; SD, standard deviation; FPL, federal poverty level; BMI, body mass index.

### 3.2 Bivariate association between insufficient sleep and sample characteristics

As shown in Table 2, there was a significant bivariate association between insufficient sleep and a number of variables. One in three (33.7%) children who were exposed to three or more ACEs compared to 26.1% of children exposed to two ACEs, 24.5% of children exposed to one ACE, and 17.3% of children not exposed to ACEs had insufficient sleep [χ^2^(3) = 550.48, *p *< .001]. The average family resilience score among children who had insufficient sleep was significantly lower than the average family resilience score among children who had sufficient sleep (*M*_had sufficient sleep_ = 13.6 vs. *M*_had insufficient sleep_ = 12.9, *t* = 20.96, *p *< .001). Respondents were more likely to have insufficient sleep if they were older, non-Hispanic black, were from households with income below the poverty level, received welfare assistance, had special health care needs, had functional difficulties, or were overweight or with obesity.

**Table 2:** Bivariate association between sample characteristics and insufficient sleep (*N* = 28,097).

Variables	Insufficient sleep		Chi-square (*p*-value)
	No	Yes	
ACEs			550.48 (*p* < .001)
None	82.7	17.3	
One ACE	75.5	24.5	
Two ACEs	73.9	26.1	
Three or more ACEs	66.3	33.7	
Age			2,037.63 (*p* < .001)
10–12 years	89.4	10.6	
13–15 years	77.2	22.8	
16–17 years	60.4	39.6	
Sex			10.89 (*p *= .160)
Male	78.4	21.6	
Female	76.8	23.2	
Race/ethnicity			257.60 (*p* < .001)
Non-Hispanic White	80.4	19.6	
Non-Hispanic Black	68.7	31.3	
Hispanic	75.7	24.3	
Other race/ethnicity	79.7	20.3	
Immigrant status			0.43 (*p* = .492)
Nonimmigrant	77.6	22.4	
Immigrant	76.9	23.1	
Household poverty level			154.48 (*p* < .001)
0–99% FPL	73.6	26.4	
100–199% FPL	74.7	25.3	
200–399% FPL	77.8	22.2	
400% FPL or greater	81.5	18.5	
Receipt of welfare assistance			68.31 (*p* = .003)
No	78.5	21.5	
Yes	72.8	27.2	
Child has special health care needs			56.18 (*p* < .001)
No	78.7	21.3	
Yes	74.3	25.7	
Functional difficulties			218.70 (*p* < .001)
None	79.7	20.3	
One functional difficulty	75.7	24.3	
Two or more functional difficulties	68.7	31.3	
BMI			105.85 (*p* < .001)
Underweight/Normal	79.0	21.0	
Overweight	77.2	22.8	
Obese	71.8	28.2	
Resilience, mean (SD)	13.6 (2.3)	12.9 (2.5)	*t* = 20.96 (*p* < .001)

ACE, adverse childhood experiences; SD, standard deviation; FPL, federal poverty level; BMI, body mass index.

### 3.3 Multivariate logistic regression results examining exposure to ACEs insufficient sleep

Table 3 shows the multivariate logistic regression results examining exposure to ACEs and insufficient sleep. Controlling for the effects of other factors in Model 2, we found a dose-response effect of exposure to ACEs on insufficient sleep. The odds of having insufficient sleep were 1.82 times higher for children exposed to three or more ACEs (AOR = 1.82, *p* < .001, 95% CI = 1.46–2.27), 1.4 times higher for children exposed to two ACEs (AOR = 1.40, *p* = .001, 95% CI = 1.15–1.72), and 1.32 times higher for children exposed to one ACE (AOR = 1.32, *p* = .002, 95% CI = 1.11–1.57), all when compared to children with no exposure to ACEs. This significant association was partially attenuated with the addition of family resilience in Model 3. Controlling for the effect of other factors and family resilience, the odds of having insufficient sleep were 1.63 times higher for children exposed to three or more ACEs (AOR = 1.63, *p* < .001, 95% CI = 1.30–2.05), 1.32 times higher for children exposed to two ACEs (AOR = 1.32, *p* = .007, 95% CI = 1.08–1.62), and 1.27 times higher for children exposed to one ACE (AOR = 1.27, *p* = .007, 95% CI = 1.07–1.51), all when compared to children with no exposure to ACEs. Family resilience partially mediated exposure to ACEs on insufficient sleep. Controlling for the effect of other factors, a one-point increase in family resilience score decreased the odds of having insufficient sleep by a factor of 12% (AOR = 0.88, *p* < .001, 95% CI = 0.86–0.91). Odds were more than fivefold higher for children ages 16–17 years to have insufficient sleep (AOR = 5.56, *p* < .001, 95% CI = 4.65–6.66), and 2.51 times higher for children ages 13–15 years to have insufficient sleep (AOR = 2.51, *p* < .001, 95% CI = 2.10–3.00), both when compared to children ages 10–12 years. The odds of having insufficient sleep were 1.70 times higher for non-Hispanic Black children (AOR = 1.70, *p* < .001, 95% CI = 1.38–2.09) and 1.29 times higher for Hispanic children (AOR = 1.29, *p* = .011, 95% CI = 1.06–1.56) when compared to their non-Hispanic White counterparts. Children were more likely to have insufficient sleep if they had two or more functional difficulties (AOR = 1.36, *p* = .011, 95% CI = 1.07–1.72) or had obesity (AOR = 1.32, *p* = .009, 95% CI = 1.07–1.62).

**Table 3:** Predictors of insufficient sleep (*N* = 28,097).

Variables	Model 1		Model 2		Model 3	
	AOR (95% C.I.)	*p*-value	AOR (95% C.I.)	*p*-value	AOR (95% C.I.)	*p*-value
Age (10–12 years)						
13–15 years	2.59 (2.16–3.11)	<.001	2.54 (2.12–3.05)	<.001	2.51 (2.10–3.00)	<.001
16–17 years	5.82 (4.84–6.99)	<.001	5.61 (4.66–6.74)	<.001	5.56 (4.65–6.66)	<.001
Sex (Male)						
Female	1.11 (0.97–1.27)	.133	1.11 (0.97–1.27)	.132	1.10 (0.96–1.25)	.167
Race/ethnicity (Non-Hispanic White)						
non-Hispanic Black	1.70 (1.38–2.08)	<.001	1.65 (1.34–2.03)	<.001	1.70 (1.38–2.09)	<.001
Hispanic	1.24 (1.02–1.50)	.032	1.25 (1.03–1.51)	.024	1.29 (1.06–1.56)	.011
Other race/ethnicity	1.09 (0.89–1.32)	.405	1.07 (0.88–1.30)	.519	1.02 (0.84–1.25)	.812
Immigrant status (Non-immigrant)						
Immigrant	0.83 (0.59–1.16)	.274	0.88 (0.62–1.24)	.455	0.90 (0.64–1.26)	.533
Household poverty level (0–99% FPL)						
100–199% FPL	1.08 (0.85–1.39)	.527	1.07 (0.83–1.37)	.595	1.14 (0.89–1.46)	.310
200–399% FPL	0.92 (0.71–1.18)	.519	0.93 (0.72–1.20)	.571	0.95 (0.74–1.23)	.714
400% FPL or greater	0.79 (0.62–1.00)	.055	0.85 (0.67–1.09)	.203	0.88 (0.68–1.12)	.287
Receipt of welfare assistance (No)						
Yes	1.07 (0.83–1.38)	.585	0.96 (0.74–1.24)	.741	1.00 (0.77–1.29)	.981
Child has special health care needs (No)						
Yes	1.03 (0.87–1.22)	.721	.97 (0.82–1.15)	.764	0.97 (0.82–1.14)	.679
Functional difficulties (None)						
One functional difficulty	1.27 (1.06–1.52)	.009	1.22 (1.02–1.46)	.032	1.18 (0.99–1.41)	.072
Two or more functional difficulties	1.58 (1.24–1.99)	<.001	1.46 (1.15–1.85)	.002	1.36 (1.07–1.72)	.011
BMI (Underweight/Normal)						
Overweight	1.12 (0.93–1.35)	.237	1.09 (0.90–1.32)	.363	1.08 (0.90–1.30)	.423
Obese	1.39 (1.13–1.71)	.002	1.33 (1.08–1.65)	.008	1.32 (1.07–1.62)	.009
ACEs (None)						
One ACE			1.32 (1.11–1.57)	.002	1.27 (1.07–1.51)	.007
Two ACEs			1.40 (1.15–1.72)	.001	1.32 (1.08–1.62)	.007
Three or more ACE			1.82 (1.46–2.27)	<.001	1.63 (1.30–2.05)	<.001
Resilience					0.88 (0.86–0.91)	<.001

NB: Reference category is indicated in bracket.

ACEs, adverse childhood experiences; SD, standard deviation; FPL, federal poverty level; BMI, body mass index.

## 4 Discussion

This study examined the association between exposure to ACEs and insufficient sleep, focusing on family resilience as a mediator. Overall, a little over one in five adolescents in our sample did not get sufficient sleep. This is lower than prior studies in which up to 75% of adolescents surveyed did not get sufficient sleep (3, 6). This difference may be attributable to the type of report—data from our study used parent-reports of adolescent sleep, whereas past studies that found higher estimates were data based on adolescent self-report (3). A prior study with elementary-age children found that children self-reported more sleep problems than their parents reported (36). Given the adverse outcomes associated with insufficient sleep among adolescents, it could be helpful for schools to include ongoing programs to educate adolescents and their families about better sleeping practices. In addition, it is possible that later school start times, as advocated by the American Physical Therapy Association, the Centers for Disease Control and Prevention, and the American Academy of Pediatrics, could facilitate improved sleep for adolescents (37).

Consistent with prior literature (9, 20, 21), we found a positive association between exposure to ACEs and insufficient sleep. Some prior studies have found that ACEs are associated with negative sleep outcomes among adults, such as shorter sleep duration/insufficient sleep, poor sleep quality, and overall sleep problems (37, 38). One recent study found that adolescents exposed to at least two ACEs were 70% more likely to have insufficient sleep than those who had no ACEs (11). This may result from the physiological impact of exposure to trauma on child well-being; indeed, prior researchers have found a link between post-traumatic stress disorder resulting from trauma and insufficient sleep among maltreated adolescents (39). As more focus is placed on screening and responding to ACEs in schools, it could be important to include sleep screenings and education on sleep health to identify potential impacts of adversity on adolescent sleep.

Building upon Lin and colleagues' (11) study on exposure to ACEs and insufficient sleep, our findings suggest that family resilience may partially mediate the association between exposure to adversity and insufficient sleep among adolescents. In a systematic review of family contextual factors and childhood sleep, Covington and colleagues (40) posited that the family context may contribute to child sleep-wake regulation; in households with less structure, routine, or discord, a child's ability to regulate their sleep could be negatively impacted. The current findings are set in a slightly different context than Covington et al.'s (40) study due to the conceptualization of family context. The context is resilience and is focused on the family's closeness and ability to work together to resolve issues. Covington et al.'s study considers a number of different factors as part of the family context, though the findings of the present study are in partial alignment with that of Covington et al. (40) that included family discord as part of their conceptualization which aligns more closely with the current study's mediator. It is also worth noting that other researchers have found sufficient child sleep to improve family functioning and harmony (41). Therefore, future research could explore this association longitudinally to determine the directionality of findings. For example, future research could use multiple measures of sleep (e.g., parent-report, biometric measures, adolescent-report) to track adolescents over time and the function of household factors such as family functioning and cohesion.

In addition to our main independent variables, we found child demographic factors such as child age, functional difficulties, and BMI were associated with insufficient sleep. Prior studies have had inconsistent findings on the association between child age and sleep; Baiden and colleagues (6) found no significant association between age and insufficient sleep among a nationally representative sample of adolescents ages 14–18 years. In contrast to some prior research (42), we did not find a significant association between child gender and insufficient sleep. It is possible that the lack of difference by gender among our sample could be due to the parental report of adolescent insufficient sleep; some prior research has found that compared to their adolescent children, parents are less likely to report risk related to sleep (43). With regards to BMI, our findings are consistent with prior research that have found BMI to be positively associated with insufficient sleep (42).

In parallel to the child factors explored above, some experts have raised concern that children of color may be at higher risk of experiencing insufficient sleep than their White peers (44). Even after adjusting for factors such as household poverty level, exposure to ACEs, and special child needs or functional limitations, children who were Black or Hispanic had higher odds of having insufficient sleep than their non-Hispanic White peers. It is possible that exposure to discrimination or racism may contribute to sleep insufficiency and sleep disturbance among adolescents (44, 45). While we found an association between race/ethnicity and insufficient sleep, immigrant status did not impact insufficient sleep among our sample of adolescents. Some prior literature has found that U.S.-born Latinos have higher rates of sleep insufficiency than immigrant Latinos (46), aligning with the immigrant paradox that posits immigrants to the U.S. tend to have better health outcomes than U.S.-born citizens (47). It is possible that the relatively low percentage of foreign-born adolescents in our sample was not sufficient to detect slight differences. However, it is also possible that after adjusting for other factors such as family resilience, ACEs, and household poverty, some of the factors that may influence the immigrant paradox were accounted for.

Surprisingly, we did not find a significant association between household poverty status or receipt of financial assistance and insufficient sleep. An international study that compared the U.S. and other countries found that individuals with financial concerns had less sleep on average than those without financial difficulties (48). Other literature has linked lower socioeconomic status to higher risk of insufficient sleep among children and adolescents (49). However, one other study using national data from the U.S. found that higher income and more highly educated parents of adolescents were more likely to report sleep insufficiency among their adolescents than lower-income parents (50). Therefore, future research could also utilize adolescent self-report and parental reports or sleep diaries to get a more accurate measure of insufficient sleep across socioeconomic statuses (51).

### 4.1 Limitations

This study has some limitations that are worth noting. First, the outcome variable, insufficient sleep was reported by parents of adolescents. Given the difference in the proportion of adolescents in our sample that reported insufficient sleep with proportions in other recent national studies using adolescent self-reported data, future research could triangulate reports and explore reasons for these discrepancies. Also, the lack of an objective sleep measure (e.g., actigraphy) and lack of additional measures of other dimensions of sleep health, such as sleep disorders, sleep quality, DSP, etc. may impact the findings reported in the current study. Future studies that employed objective sleep measures or other dimensions of sleep health are warranted. Second, the data used in this study are cross-sectional. Therefore, we are unable to determine directionality for our findings—particularly with regard to family resilience. As we found evidence of shared variance between family resilience and ACEs in our model, future research is needed to follow adolescents longitudinally and determine directionality. Third, the ACE items employed in this study were based on parent reports. Therefore, it is possible that some items were underreported due to social desirability. Also, it is possible that older adolescents may have experienced ACEs that parents are not aware of, further highlighting the need for adolescent self-report. The items asked in this database also differ slightly from items asked in the CDC ACE module and in the original Kaiser Permanente ACE study. While we can compare our findings with other studies using NSCH, these differences in operationalization limit our ability to directly compare with ACE studies that use other data sources.

## 5 Conclusion

This study examined the association between exposure to ACEs and insufficient sleep among adolescents, exploring the role of family resilience as a mediator. Although exposure to ACEs was positively associated with insufficient sleep among adolescents, family resilience partially mediates the association. Therefore, there are modifiable factors that may improve sleep outcomes among adolescents who have been exposed to adversity. Future research can help elucidate findings and establish the directionality of this association.

## Data Availability

Publicly available datasets were analyzed in this study. This data can be found here: https://www.childhealthdata.org/Learn-about-the-nsch/NSCH.

## References

[1] Owens J Adolescent Sleep Working Group, Committee on Adolescence Au R Carskadon M Millman R Insufficient sleep in adolescents and young adults: an update on causes and consequences. Pediatrics. (2014) 134(3):e921–32. 10.1542/peds.2014-1696 25157012 PMC8194472

[2] Chaput J-P Dutil C Sampasa-Kanyinga H. Sleeping hours: what is the ideal number and how does age impact this? Nat Sci Sleep. (2018) 10:421–30. 10.2147/NSS.S163071 30568521 PMC6267703

[3] Kann L McManus T Harris WA Shanklin SL Flint KH Queen B Youth risk behavior surveillance—United States, 2017. MMWR Surveill Summ. (2018) 67(8):1–114. 10.15585/mmwr.ss6708a1 29902162 PMC6002027

[4] Quante M Khandpur N Kontos EZ Bakker JP Owens JA Redline S. “Let’s talk about sleep”: a qualitative examination of levers for promoting healthy sleep among sleep-deprived vulnerable adolescents. Sleep Med. (2019) 60:81–8. 10.1016/j.sleep.2018.10.044 30606643 PMC6571071

[5] Sampasa-Kanyinga H Lien A Hamilton HA Chaput J-P. Cyberbullying involvement and short sleep duration among adolescents. Sleep Health. (2022) 8(2):183–90. 10.1016/j.sleh.2021.11.009 35120851

[6] Baiden P Tadeo SK Peters KE. The association between excessive screen-time behaviors and insufficient sleep among adolescents: findings from the 2017 youth risk behavior surveillance system. Psychiatry Res. (2019) 281:112586. 10.1016/j.psychres.2019.112586 31629305

[7] Walsh F. Applying a family resilience framework in training, practice, and research: mastering the art of the possible. Fam Process. (2016) 55(4):616–32. 10.1111/famp.12260 27921306

[8] Gordon AM Carrillo B Barnes CM. Sleep and social relationships in healthy populations: a systematic review. Sleep Med Rev. (2021) 57:101428. 10.1016/j.smrv.2021.101428 33596514

[9] Duraccio K Erickson L Jones MS Pierce H. Early adverse childhood experiences and adolescent sleep outcomes. Child Abuse Negl. (2024) 147:106593. 10.1016/j.chiabu.2023.106593 38061279

[10] Rojo-Wissar DM Sosnowski DW Ingram MM Jackson CL Maher BS lfano CA Associations of adverse childhood experiences with adolescent total sleep time, social jetlag, and insomnia symptoms. Sleep Med. (2021) 88:104–15. 10.1016/j.sleep.2021.10.019 34742038 PMC9012105

[11] Lin SX Bresnahan M Amsel L McReynolds L Cheslack-Postava K Hoven CW. Adverse childhood experiences (aces) and insufficient sleep among US children and adolescents. Acad Pediatr. (2022) 22(6):965–71. 10.1016/j.acap.2022.02.007 35167994 PMC9484003

[12] Waehrer GM Miller TR Silverio Marques SC Oh DL Burke Harris N. Disease burden of adverse childhood experiences across 14 states. PLoS One. (2020) 15(1):e0226134. 10.1371/journal.pone.0226134 31990910 PMC6986706

[13] Mansuri F Nash MC Bakour C Kip K. Adverse childhood experiences (ACEs) and headaches among children: a cross-sectional analysis. Headache J Head Face Pain. (2020) 60(4):735–44. 10.1111/head.13773 32065390

[14] Santos M Burton ET Cadieux A Gaffka B Shaffer L Cook JL Adverse childhood experiences, health behaviors, and associations with obesity among youth in the United States. Behav Med. (2023) 49(4):381–91. 10.1080/08964289.2022.2077294 35792894

[15] Bethell CD Gombojav N Whitaker RC. Family resilience and connection promote flourishing among US children, even amid adversity. Health Aff (Millwood). (2019) 38(5):729–37. 10.1377/hlthaff.2018.05425 31059374

[16] Uddin J Alharbi N Uddin H Hossain MB Hatipoglu SS Long DL Parenting stress and family resilience affect the association of adverse childhood experiences with children’s mental health and attention-deficit/hyperactivity disorder. J Affect Disord. (2020) 272:104–9. 10.1016/j.jad.2020.03.132 32379600

[17] Song J Fogarty K Suk R Gillen M. Behavioral and mental health problems in adolescents with ADHD: exploring the role of family resilience. J Affect Disord. (2021) 294:450–8. 10.1016/j.jad.2021.07.073 34325164

[18] Herbell K Breitenstein SM Melnyk BM Guo J. Family resilience and flourishment: well-being among children with mental, emotional, and behavioral disorders. Res Nurs Health. (2020) 43(5):465–77. 10.1002/nur.22066 32797699

[19] Gómez A. Associations between family resilience and health outcomes among kinship caregivers and their children. Child Youth Serv Rev. (2021) 127:106103. 10.1016/j.childyouth.2021.106103

[20] Sivertsen B Harvey AG Pallesen S Hysing M. Mental health problems in adolescents with delayed sleep phase: results from a large population-based study in Norway. J Sleep Res. (2015) 24(1):11–8. 10.1111/jsr.12254 25358244

[21] Chang L-Y Wu C-C Yen L-L Chang H-Y. The effects of family dysfunction trajectories during childhood and early adolescence on sleep quality during late adolescence: resilience as a mediator. Soc Sci Med. (2019) 222:162–70. 10.1016/j.socscimed.2019.01.010 30641286

[22] Ríos-Salas V Larson A. Perceived discrimination, socioeconomic status, and mental health among latino adolescents in US immigrant families. Child Youth Serv Rev. (2015) 56:116–25. 10.1016/j.childyouth.2015.07.011

[23] Jackson CL Hu FB Redline S Williams DR Mattei J Kawachi I. Racial/ethnic disparities in short sleep duration by occupation: the contribution of immigrant status. Soc Sci Med. (2014) 118:71–9. 10.1016/j.socscimed.2014.07.059 25108693 PMC4744798

[24] Caballero TM Johnson SB Buchanan CRM DeCamp LR. Adverse childhood experiences among hispanic children in immigrant families versus US-native families. Pediatrics. (2017) 140(5):1–10. 10.1542/peds.2017-0297 28993445

[25] Motti-Stefanidi F Masten AS. A resilience perspective on immigrant youth adaptation and development. In: Cabrera N Leyendecker B, editors. Handbook on Positive Development of Minority Children and Youth. Cham, Switzerland: Springer (2017). p. 19–34.

[26] Sheehan C Powers D Margerison-Zilko C McDevitt T Cubbin C. Historical neighborhood poverty trajectories and child sleep. Sleep Health. (2018) 4(2):127–34. 10.1016/j.sleh.2017.12.005 29555124 PMC5863576

[27] Wiggs L France K. Behavioural treatments for sleep problems in children and adolescents with physical illness, psychological problems or intellectual disabilities. Sleep Med Rev. (2000) 4(3):299–314. 10.1053/smrv.1999.0094 12531171

[28] Miller MA Kruisbrink M Wallace J Ji C Cappuccio FP. Sleep duration and incidence of obesity in infants, children, and adolescents: a systematic review and meta-analysis of prospective studies. Sleep. (2018) 41(4):1–19. 10.1093/sleep/zsy018 29401314

[29] Child and Adolescent Health Measurement Initiative (CAHMI). 2017–2018 National Survey of Children’s Health (2 Years Combined Data Set): SPSS Data Set. Data Resource Center for Child and Adolescent Health supported by Cooperative Agreement U59MC27866 from the U.S. Department of Health and Human Services, Health Resources and Services Administration (HRSA). Maternal and Child Health Bureau (MCHB). (2019). Available online at: childhealthdata.org (accessed: February 13, 2020)

[30] Ghandour RM Jones JR Lebrun-Harris LA Minnaert J Blumberg SJ Fields J The design and implementation of the 2016 national survey of children’s health. Matern Child Health J. (2018) 22(8):1093–102. 10.1007/s10995-018-2526-x 29744710 PMC6372340

[31] Baiden P LaBrenz CA Thrasher S Asiedua-Baiden G Harerimana B. Adverse childhood experiences and household food insecurity among children aged 0–5 years in the USA. Public Health Nutr. (2021) 24(8):2123–31. 10.1017/S1368980020002761 32867879 PMC10195571

[32] Baiden P LaBrenz CA Okine L Thrasher S Asiedua-Baiden G. The toxic duo: bullying involvement and adverse childhood experiences as factors associated with school disengagement among children. Child Youth Serv Rev. (2020) 119C:105383. 10.1016/j.childyouth.2020.105383

[33] Panisch LS Baiden P Findley E Jahan N LaBrenz CA. Examining the association between adverse childhood experiences and asthma among children in the United States: the intersection of sex and race/ethnicity. J Asthma. (2022) 59(6):1122–30. 10.1080/02770903.2021.1910296 33783306

[34] American Academy of Sleep Medicine. Recharge with Sleep: Pediatric Sleep Recommendations Promoting Optimal Health. (2016). Available online at: https://aasm.org/recharge-with-sleep-pediatric-sleep-recommendations-promoting-optimal-health/ (Accessed March 3, 2022).

[35] Jackson DB Chilton M Johnson KR Vaughn MG. Adverse childhood experiences and household food insecurity: findings from the 2016 national survey of children’s health. Am J Prev Med. (2019) 57(5):667–74. 10.1016/j.amepre.2019.06.004 31522923

[36] Owens JA Spirito A McGuinn M Nobile C. Sleep habits and sleep disturbance in elementary school-aged children. J Dev Behav Pediatr. (2000) 21(1):27–36. 10.1097/00004703-200002000-00005 10706346

[37] Knopf A. Most middle/high school students don’t get enough sleep: CDC. Brown Univ Child Adolesc Behav Lett. (2018) 34(3):4–4. 10.1002/cbl.30280

[38] Charles LE Mnatsakanova A Fekedulegn D Violanti JM Gu JK Andrew ME. Associations of adverse childhood experiences (ACEs) with sleep duration and quality: the BCOPS study. Sleep Med. (2022) 89:166–75. 10.1016/j.sleep.2021.12.011 35026653 PMC8916064

[39] Brown TSH Belcher HM Accardo J Minhas R Briggs EC. Trauma exposure and sleep disturbance in a sample of youth from the national child traumatic stress network core data set. Sleep Health. (2016) 2(2):123–8. 10.1016/j.sleh.2016.03.001 28923254

[40] Covington LB Patterson F Hale LE Teti DM Cordova A Mayberry S The contributory role of the family context in early childhood sleep health: a systematic review. Sleep Health. (2021) 7(2):254–65. 10.1016/j.sleh.2020.11.010 33436342

[41] Muller D Paine S-J Wu LJ Signal TL. “Their sleep means more harmony”: maternal perspectives and experiences of preschoolers’ sleep in ethnically and socioeconomically diverse families in Aotearoa/New Zealand. Qual Health Res. (2019) 29(14):2023–34. 10.1177/1049732319842156 30973062

[42] Tambalis KD Panagiotakos DB Psarra G Sidossis LS. Insufficient sleep duration is associated with dietary habits, screen time, and obesity in children. J Clin Sleep Med. (2018) 14(10):1689–96. 10.5664/jcsm.7374 30353810 PMC6175799

[43] Gersh E Richardson LP Katzman K Spielvogle H Arghira AC Zhou C Adolescent health risk behaviors: parental concern and concordance between parent and adolescent reports. Acad Pediatr. (2018) 18(1):66–72. 10.1016/j.acap.2017.08.012 28870652

[44] Johnson DA Billings ME Hale L. Environmental determinants of insufficient sleep and sleep disorders: implications for population health. Curr Epidemiol Rep. (2018) 5(2):61–9. 10.1007/s40471-018-0139-y 29984131 PMC6033330

[45] Xie M Yip T Cham H El-Sheikh M. The impact of daily discrimination on sleep/wake problem trajectories among diverse adolescents. Child Dev. (2021) 92(5):e1061–74. 10.1111/cdev.13605 34106461 PMC11174140

[46] Miguez MJ Bueno D Perez C. Disparities in sleep health among adolescents: the role of sex, age, and migration. Sleep Disord. (2020) 2020:1–6. 10.1155/2020/5316364 PMC7024093 32089893

[47] LaBrenz CA Panisch LS Lawson J Borcyk AL Gerlach B Tennant PS Adverse childhood experiences and outcomes among at-risk Spanish-speaking Latino families. J Child Fam Stud. (2020) 29(5):1221–35. 10.1007/s10826-019-01589-0

[48] Hafner M Stepanek M Taylor J Troxel WM Van Stolk C. Why sleep matters—the economic costs of insufficient sleep: a cross-country comparative analysis. Rand Health Q. (2017) 6(4):11. ; 28983434 10.7249/rhq-06-04-11 PMC5627640

[49] Chang L-Y Chiang T. Association between socioeconomic status and the trajectory of insufficient sleep: maternal emotional support as a moderator. Soc Sci Med. (2020) 261:113237. 10.1016/j.socscimed.2020.113237 32745826

[50] Hawkins SS Takeuchi DT. Social determinants of inadequate sleep in US children and adolescents. Public Health. (2016) 138:119–26. 10.1016/j.puhe.2016.03.036 27207726 PMC5012907

[51] Reed DL Sacco WP. Measuring sleep efficiency: what should the denominator be? J Clin Sleep Med. (2016) 12(2):263–6. 10.5664/jcsm.5498 26194727 PMC4751425

